# Functional central nervous system regeneration: Challenges from axons to circuits

**DOI:** 10.4103/NRR.NRR-D-24-01633

**Published:** 2025-04-29

**Authors:** Apolline Delaunay, Mickaël Le Boulc’h, Stephane Belin, Homaira Nawabi

**Affiliations:** Univ. Grenoble Alpes, Inserm, U1216, Grenoble Institut Neurosciences, Grenoble, France

The mature central nervous system (CNS, composed of the brain, spinal cord, olfactory and optic nerves) is unable to regenerate spontaneously after an insult, both in the cases of neurodegenerative diseases (for example Alzheimer’s or Parkinson’s disease) or traumatic injuries (such as spinal cord lesions). In the last 20 years, the field has made significant progress in unlocking axon regrowth. The lesion’s environment has been well characterized, notably the contribution of the glial scar and myelin debris-associated molecules to axon regeneration inhibition. Surprisingly, the modulation of these factors only promotes limited axon regrowth (Varadarajan et al., 2022). Thus, the focus has progressively shifted from the environment of the injured axons to the neurons themselves. Several regenerative models have been developed targeting different steps of gene expression from epigenetics to translation processes. For example, regarding transcription regulation, it has been found that the modulation of transcription factors, such as suppressor of cytokine signaling 3, a negative regulator of Janus kinases/signal transducers and activators of transcription pathway, the Krüppel-like factors family or c-myc could promote axon regeneration (Varadarajan et al., 2022). Activating the mammalian target of rapamycin pathway, a major regulator of protein translation also promotes regeneration (Varadarajan et al., 2022). Moreover, combinatorial approaches such as activation of mammalian target of rapamycin, Janus kinases/signal transducers and activators of transcription pathways along with c-myc overexpression lead axon regeneration up to several millimeters from the eye to the brain (Belin et al., 2015). Even if these results are very exciting, functional recovery remains a pending issue. Indeed, recent work highlights that regrowing axons is not the only aspect necessary for circuit formation and several unexpected roadblocks have been uncovered: (i) the majority of regenerating axons do not reach their proper targets, (ii) nor do they regularly form synapses when they do reach their targets, and (iii) myelination in a regenerative context is still poorly characterized. In this article, we will discuss these challenges.

**Circuit formation:** Most neuronal circuits are formed during the embryonic period. Intriguingly, some tracts such as the corticospinal tract end their growth only at post-natal stages. Each phase of circuit formation is tightly regulated, especially regarding axon navigation. Once the growth period is over, the leading edge of extending axons (namely the growth cones) transforms into synapses. From this point on, topographical information carried by guidance cues, among others, is not needed. Therefore, it was unclear whether these guidance cues were still expressed in the mature nervous system and, if so, whether their expression pattern and their effects on mature axons remained the same as during development. In the injured CNS, the expression of these proteins has been mainly studied at the lesion site. Their lesion-induced overexpression has been associated with the failure of regeneration (Varadarajan et al., 2022). Interestingly, the rise of long-distance regeneration models opens up the possibility to study their contribution to mature CNS circuit formation. Vilallongue et al. (2022) showed that guidance molecules were still expressed in visual recipient nuclei of the mature brain. Interestingly most of these identified cues have been described as repulsive during CNS development. This repulsive environment of the mature CNS might play a role in the maintenance of structured circuits. As their expression remains stable in the brain when comparing pre- and post-lesion, they could prevent regenerating axons from reaching their proper target (**Figure 1A**). Recently, Delpech et al. (2024) combined a long-distance optic nerve regeneration model from Belin et al. (2015) with axon guidance modulation to address the reinnervation of the suprachiasmatic nucleus (SCN). They showed, as expected from Vilallongue et al. (2022), that the mature SCN exerts a repulsive activity on regenerating axons and identified the Slit family of guidance cues as one of the effectors. The inhibition of their Robo receptors within regenerating RGC axons led to SCN innervation (**Figure 1A**). This newly formed circuit is functional at the cellular level as light exposure induces specific expression of the immediate early gene c-fos in the SCN. With regard to circuit functionality, SCN reinnervation led to a partial restoration of the circadian rhythm. This work also highlights new hurdles: while the intact SCN receives optic nerve innervation from the ventral side and synapses mainly form in the core of the nuclei, regenerating axons invade the SCN from the dorsal side and form synapses with the SCN shell. This differential innervation could contribute to the partial recovery of function. Moreover, the rabies tracing experiment revealed that RGC that normally avoid the SCN, such as alpha-RGC, enter this nucleus in the context of regeneration. It would be interesting to modulate Robo/Slit in specific subpopulations of neurons to control circuit formation more specifically. The contribution of guidance signaling has been also reported in the context of spinal cord injuries. It has been shown that the growth factor glial-cell-line-derived neurotrophic factor delivered by biomaterial depots acts as a chemoattractive cue to help regenerating axons to cross the lesion site (Anderson et al., 2018). These studies suggest that controlling the guidance landscape of regenerative axons should be considered to achieve proper connectivity sustaining functional recovery.

**Figure 1:**
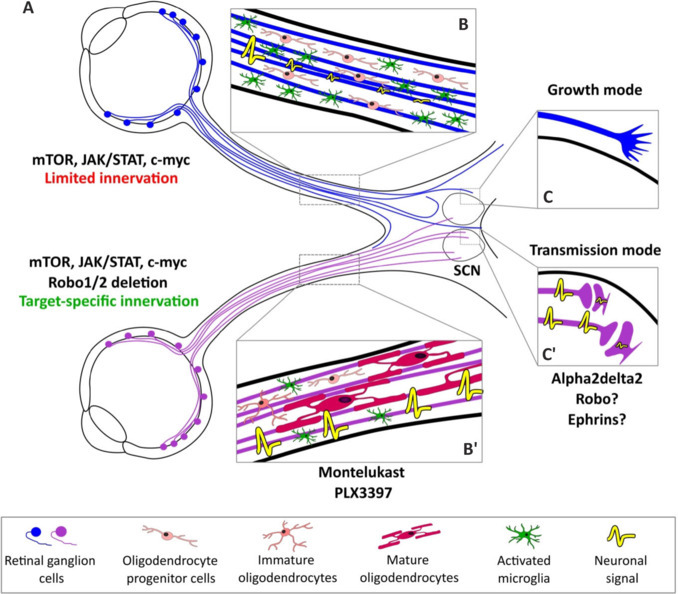
Target connectivity, synaptogenesis, and myelination: three pillars sustaining functional recovery after a central nervous system lesion. Schematic showing the key steps to achieve functional circuit: (A) Regenerating axons (in blue) have limited ability to innervate brain targets as they are highly repulsive. Modulation of these repulsive factors, such as Robo/Slit, leads to specific innervation of the SCN (purple axons). (B-B’) Enhancing oligodendrocytes maturation through the combined inhibition of GPR17 (by Montelukast) and microglia (by PLX3397) leads to increased myelination of regenerating axons. (C-C’) In the regenerative context, the alpha2delta2 subunit of voltage-gated calcium channels acts as a positive regulator for synapse formation by switching axons from a growth to a transmission state. Other cues are also involved in synapse formation and maintenance (for example Robo and Ephrins). Created with Inkscape software (v 1.3). JAK: Janus kinases; mTOR: mammalian target of rapamycin; SCN: suprachiasmatic nucleus; STAT: signal transducers and activators of transcription.

**Synapse formation and maturation:** Most models of regeneration only report a partial recovery of function. These observations strongly suggest the potential impairment of regenerating axons to integrate functionally into neuronal circuits. One key aspect is the neuron-to-partner communication through synapses between the regenerating axons and their targets. Several studies assessed synaptogenesis in the SCN following RGC regeneration (Li et al., 2015; Delpech et al., 2024). The colocalization of presynaptic proteins (namely vesicular glutamate transporter 2) and protein of the postsynaptic density (PSD95) strongly suggest the ability of regenerating axons to make synapses with their targeted neurons. The functionality of these synapses was confirmed using the expression of the immediate early gene *c-fos* in the SCN upon RGC stimulation using optogenetics or light. Li et al. (2015) were even able to record, *ex vivo*, evoked excitatory postsynaptic current in the SCN following optic chiasm stimulation. However, the number of c-fos expressing neurons and the amplitude of the excitatory postsynaptic current remain lower than in intact mice. More recently, Delpech et al. (2024) showed that regenerating axons can make functional synapses with SCN neurons. These results are encouraging as they show that synaptogenesis happens spontaneously in regenerating conditions. They also suggest that the newly formed synapses might be too few and/or too immature to generate synaptic inputs comparable to normal conditions leading to partial functional recovery. It is not clear to what extent regenerating axons can switch from a growth state (axons with a growth cone) to a transmission one (axons with a synapse) and whether this ability could contribute to circuit formation. Tedeschi et al., (2016) showed that promoting synapse formation by overexpressing the Alpha2delta2 subunit of voltage-gated calcium channels led to axon growth inhibition (**Figure 1C-C’**). In contrast, blocking synapse formation enhances axon elongation. Therefore, there is a tight balance between synapse formation and axon growth. This study highlights that controlling the timing and the location of synapse formation is crucial for circuit formation during regeneration. Synaptogenesis also seems to be controlled by guidance cues. In the hippocampus, for example, the receptor Robo2 is required in CA1 pyramidal excitatory synapse formation but not the inhibitory ones (Blockus et al., 2021). Moreover, their function appears to be compartmentalized in neurons as Robo2 controls the formation of excitatory synapses in proximal but not distal dendrite regions (Blockus et al., 2021). Deletion of Robo in CA1 neurons induces a significantly reduced event frequency during behavioral tests (treadmill running), which is linked to a reduction of the firing rate of hippocampal pyramidal neurons involved in spatial processing, highlighting its role both in guidance and synaptogenesis (**Figure 1C’**). Ephrins have also been implicated in inhibitory synapse regulation in parvalbumin interneurons: overexpression of astrocytic ephrin-B1 increases parvalbumin-expressing neurons and pyramidal cells connectivity, most likely due to an increase in synapse number (Sutley-Koury et al., 2024; **Figure 1C’**). The deletion of Ephrin-B1 leads to an increase in PTZ-induced seizure number and their severity. This observation is consistent with the hypothesis that impaired inhibition favors the development of hyperactive neuronal networks in autism spectrum disorder and epilepsy. Thus, regarding the critical role of synaptogenesis for functional recovery, it is a key milestone in future research for CNS regeneration.

**Myelination:** A final challenge to reach functional recovery is the neuron’s ability to propagate electrical signals through action potentials. Therefore, myelination appears to be a critical step. This process involves mechanisms related to neurons themselves but also to other cell types, namely the oligodendrocytes or microglia. This need to myelinate regrowing axons has been brought to light by Bei et al. (2016) and other labs. They used a short-distance regeneration model in which the lesion was performed just before the entry of RGC axons to the superior colliculus. Even if regenerating RGC axons entered the superior colliculus and formed synapses, functional recovery remained poor. This result was correlated with an absence of regenerating axon myelination, thus affecting their conductivity (**Figure 1B**). To circumvent this lack of conductivity, authors locally applied the voltage-gated potassium channel blocker 4-aminopyridine (known to improve the conduction of unmyelinated axons in cases of multiple sclerosis). This treatment significantly increased the amplitude of the local field potential in the superior colliculus compared to control, improving vision restoration. Surprisingly, the improvement in conductivity and behavior was only transient. One possible explanation for this is that pharmacological approaches to improve axon conductivity might not be sufficient in the long term. Therefore, proper myelination of regenerating axons should be addressed. It has been shown that the axon’s initial segment, the excitable domains of axons (along with the nodes of Ranvier), plays a critical role during myelination. Upon optic nerve injury, the axon’s initial segment is disrupted. When axons of the optic nerve model are regenerating, the axon’s initial segment is rebuilt starting from the retina towards the distal end of the optic nerve. However, the myelination process slows down as the distance from the retina increases, resulting in its impairment (Marin et al., 2016). Wang et al. (2020) further address regenerating axon myelination. Oligodendrocytes are the main actors of myelination in the CNS. Oligodendrocyte precursor cells undergo transient proliferation after an optic nerve lesion but fail to differentiate into mature myelination-competent oligodendrocytes. This leads to a myelination deficit that exposes potassium channels otherwise located under the myelin sheath. To overcome this, they locally applied Montelukast, an approved drug known to promote oligodendrocyte precursor cell differentiation acting as an antagonist of leukotriene receptors including GPR17. Interestingly, GPR17 expression is very low in the mature CNS but its expression is triggered by axon lesion and is restricted to oligodendrocytes. Montelukast treatment promotes early differentiation of oligodendrocyte precursor cells, however, the myelination of regenerated axons remained modest (20% of axons) due to a poor maturation of differentiated oligodendrocytes (**Figure 1B’**). Surprisingly, oligodendrocyte maturation is inhibited by microglia. Therefore, authors combined Montelukast with PLX3397 (an inhibitor of microglia), leading to a further increase of myelination of regenerating axons (up to 60%) (**Figure 1B’**). This experiment shows that multiple regulatory mechanisms are at play to achieve myelination of regenerating axons. It is a multifactorial process that needs further analysis to be fully understood. Yet, many questions remain regarding myelination: are these mechanisms, described in the optic nerve, shared with axons within the spinal cord or the brain? What would be the best time to treat patients and finally how long should these treatments last?

**Concluding remarks:** Even if the field of axon regeneration has made spectacular progress in the last 20 years, many gaps remain to be filled. Indeed, circuit formation is more complicated than what was originally thought. Hence, target connectivity, synaptogenesis, and myelination appear as the three pillars sustaining functional recovery. Future development of therapeutic approaches requires seizing this complexity and the use of multimodal approaches seems to be a plausible solution to overcome the failure of CNS regeneration.

*We apologize to colleagues whose work we could not cite due to space limitations.*

*This work was supported by ANR (ANR-21-CE16-0008-01) and UNADEV (A22018CS) (to HN), ANR (ANR-21-CE16-0008-02 and ANR-23-CE52-0007) and UNADEV (A22020CS) (to SB).*

## References

[R1] Anderson MA, O’Shea TM, Burda JE, Ao Y, Barlatey SL, Bernstein AM, Kim JH, James ND, Rogers A, Kato B, Wollenberg AL, Kawaguchi R, Coppola G, Wang C, Deming TJ, He Z, Courtine G, Sofroniew MV (2018). Required growth facilitators propel axon regeneration across complete spinal cord injury. Nature.

[R2] Bei F, Lee HHC, Liu X, Gunner G, Jin H, Ma L, Wang C, Hou L, Hensch TK, Frank E, Sanes JR, Chen C, Fagiolini M, He Z (2016). Restoration of visual function by enhancing conduction in regenerated axons. Cell.

[R3] Belin S, Nawabi H, Wang C, Tang S, Latremoliere A, Warren P, Schorle H, Uncu C, Woolf CJ, He Z, Steen JA (2015). Injury-induced decline of intrinsic regenerative ability revealed by quantitative proteomics. Neuron.

[R4] Blockus H, Rolotti SV, Szoboszlay M, Peze-Heidsieck E, Ming T, Schroeder A, Apostolo N, Vennekens KM, Katsamba PS, Bahna F, Mannepalli S, Ahlsen G, Honig B, Shapiro L, Wit J de, Losonczy A, Polleux F (2021). Synaptogenic activity of the axon guidance molecule Robo2 underlies hippocampal circuit function. Cell Rep.

[R5] Delpech C, Schaeffer J, Vilallongue N, Delaunay A, Benadjal A, Blot B, Excoffier B, Plissonnier E, Gascon E, Albert F, Paccard A, Saintpierre A, Gasnier C, Zagar Y, Castellani V, Belin S, Chédotal A, Nawabi H (2024). Axon guidance during mouse central nervous system regeneration is required for specific brain innervation. Dev Cell.

[R6] Li S, He Q, Wang H, Tang X, Ho KW, Gao X, Zhang Q, Shen Y, Cheung A, Wong F, Wong YH, Ip NY, Jiang L, Yung WH, Liu K (2015). Injured adult retinal axons with Pten and Socs3 co-deletion reform active synapses with suprachiasmatic neurons. Neurobiol Dis.

[R7] Marin MA, de Lima S, Gilbert HY, Giger RJ, Benowitz L, Rasband MN (2016). Reassembly of excitable domains after CNS axon regeneration. J Neurosci.

[R8] Sutley-Koury SN, Taitano-Johnson C, Kulinich AO, Farooq N, Wagner VA, Robles M, Hickmott PW, Santhakumar V, Mimche PN, Ethell IM (2024). EphB2 signaling is implicated in astrocyte-mediated parvalbumin inhibitory synapse development. J Neurosci.

[R9] Tedeschi A, Dupraz S, Laskowski CJ, Xue J, Ulas T, Beyer M, Schultze JL, Bradke F (2016). The calcium channel subunit Alpha2delta2 suppresses axon regeneration in the adult CNS. Neuron.

[R10] Varadarajan SG, Hunyara JL, Hamilton NR, Kolodkin AL, Huberman AD (2022). Central nervous system regeneration. Cell.

[R11] Vilallongue N, Schaeffer J, Hesse AM, Delpech C, Blot B, Paccard A, Plissonnier E, Excoffier B, Couté Y, Belin S, Nawabi H (2022). Guidance landscapes unveiled by quantitative proteomics to control reinnervation in adult visual system. Nat Commun.

[R12] Wang J, He X, Meng H, Li Y, Dmitriev P, Tian F, Page JC, Lu QR, He Z (2020). Robust myelination of regenerated axons induced by combined manipulations of GPR17 and microglia. Neuron.

